# Breakpoint Analysis for the COVID-19 Pandemic and Its Effect on the Stock Markets

**DOI:** 10.3390/e23010100

**Published:** 2021-01-12

**Authors:** Karime Chahuán-Jiménez, Rolando Rubilar, Hanns de la Fuente-Mella, Víctor Leiva

**Affiliations:** 1 Escuela de Auditoría, Centro de Investigación en Negocios y Gestión Empresarial, Facultad de Ciencias Económicas y Administrativas, Universidad de Valparaíso, Valparaíso 2362735, Chile; karime.chahuan@uv.cl; 2 Instituto de Estadística, Facultad de Ciencias, Universidad de Valparaíso, Valparaíso 2360102, Chile; rolando.rubilar@uv.cl; 3 Escuela de Comercio, Facultad de Ciencias Económicas y Administrativas, Pontificia Universidad Católica de Valparaíso, Valparaíso 2340031, Chile; hanns.delafuente@pucv.cl; 4 Escuela de Ingeniería Industrial, Facultad de Ingeniería, Pontificia Universidad Católica de Valparaíso, Valparaíso 2362807, Chile

**Keywords:** data science, econometric models, financial contagion and crisis, statistical modeling

## Abstract

In this research, statistical models are formulated to study the effect of the health crisis arising from COVID-19 in global markets. Breakpoints in the price series of stock indexes are considered. Such indexes are used as an approximation of the stock markets in different countries, taking into account that they are indicative of these markets because of their composition. The main results obtained in this investigation highlight that countries with better institutional and economic conditions are less affected by the pandemic. In addition, the effect of the health index in the models is associated with their non-significant parameters. This is due to that the health index used in the modeling would not determine the different capacities of the countries analyzed to respond efficiently to the pandemic effect. Therefore, the contagion is the preponderant factor when analyzing the structural breakdown that occurred in the world economy.

## 1. Introduction

From a financial point of view, the available data with respect to the effects of health pandemics on global market are limited [1]. The financial contagion provides evidence of self-excitation in both the United States (US) and other world markets as well as of asymmetric cross-excitation [2]. Thus, the US market generally has more influence on the jump intensity from other markets than vice versa.

Outbreaks of infectious diseases, such as SARS, attract substantial attention from a health perspective [3]. In addition, there is awareness that their economic impact can be potentially high. However, the precise mechanism of how this impact occurs is unclear.

Global trade is a mechanism through which geographically distant epidemics could affect the countries. In terms of infectious diseases, some studies have found that epidemics can interfere with short-term economic results by changing expectations and deterring investment. Nevertheless, a link between epidemics and long-term growth has not been established [4]. A study indicates that US exports and the jobs supported by such exports were negatively affected by the 2014 Ebola outbreak in West Africa [4].

Extreme events have negative multidimensional impacts on health from the economic and social point of view, particularly at the macroeconomic level [5]. Some authors [6] mentioned that econometric models allow the estimation of probable costs and benefits of a pandemic.

The immediate economic costs of epidemics in affected regions can be considerable and are therefore frequently evaluated after notable outbreaks [4]. According to [7], the risk of COVID-19 is perceived differently over time and represents the probability of being viewed as an economic crisis. This study [7] offered several important implications and endorsements for policy makers and for asset managers as the COVID-19 pandemic outbreak continues its tremendous spread in the US, causing unprecedented volatility effects on the market [8]. A country affected by a pandemic-level infection with a better-organized producer has a greater possibility of being in a better position to avoid a decrease in production [9,10]. In addition, we must mention the negative impact of COVID-19 on the financial markets, which is more marked than its positive impact. This stylized fact, also known as volatility asymmetry, has been modeled in [11,12] by means of the volatility feedback effect.

Financial markets experience crises differently, regardless of whether these crises are contagious or not. However, after the crises, appropriate models for describing unusual events leading to such crises have become essential in the fields of finance and risk management [13,14]. The literature has related global financial crises with the concepts of interconnection, contagion, and spillover effects in markets [8].

Economic expectative suggests that, when two economies are well integrated through trade, investment, and financial relationships [15], a crisis in one economy is likely to quickly spread to the other one. This because exposure to financial globalization can lead to a higher vulnerability to a financial crisis, associating them with the concept of contagion effect. The capital benefit is greater in developing markets than in developed stock markets due to investing in emerging markets tends to be riskier [16].

Different effects have been identified on volatility in the short, medium, and long terms [17]. Impacts of the factors that contribute to contagion effects of volatility have no systematic patterns. Nevertheless, if the country is the transmitter or receiver of volatility, spillovers could be a potential reason, but the interdependence of the global stock markets highly reduces the risk avoidance benefits of investing in multiple stock markets. Furthermore, a clear understanding of intensity and variations in the transmission of volatility among markets could be crucial for international risk management in terms of global coverage strategies and asset allocation decision-making. Some authors [18] mentioned that volatility with respect to financial products could measure the fluctuations of a financial security price around its expected value. Investment decisions in financial markets strongly depend on expectations regarding expected returns and asset volatility.

The literature basically distinguishes three mechanisms through which economic disturbances spread among countries [19,20,21,22]. The first mechanism corresponds to the global shocks that affect all or almost all of the countries of the world. The transmission of shocks among countries translates into increases in correlation beyond any fundamental link, which is known as excess joint movement. The second mechanism corresponds to shocks from related countries, a phenomenon that has been referred to as foundation-based contagion. The third mechanism corresponds to the contagion, which is defined as a residual element, and therefore it is related to the extent and magnitude of the international transmission of crises [23].

The transition from the calm to the turbulent regime is driven by changes in the global jump risk [23]. This suggests that the contagion of the global financial market significantly affects the price of sovereign credit risk. Therefore, neglecting financial contagion and feedback effects of sovereign credit risk on national economic and financial developments lead to spurious results with respect to the determinants of sovereign country default spreads (CDS). Regression models of CDS on variables that capture the fundamental macroeconomic determinants of this spread can be formulated [24].

The financial contagion is modeled as an equilibrium phenomenon given that liquidity preference shocks are imperfectly correlated between regions. In the face of this type of financial effect, policy makers, investors, and researchers have raised the questions of why this phenomenon occurs and what can be done to mitigate the risk of contagious crises in the future [25,26]. Failure to consider financial contagion and country risk, measured through sovereign CDS [23], could generate a national economic and financial evolution with spurious results depending on the determinants of sovereign CDS.

Separating contagion from globalization is econometrically difficult [15,27]. According to [20], a contagion grows significantly in the links among markets after a shock in a country (or a group of countries). A number of authors [20,28] pointed out that (i) if two stock markets exhibit a high level of co-movement [29]; or (ii) if a particular index increases and another index also increases; or (iii) if a particular index decreases and another index also decreases during lulls, then a high correlation after a shock in one of the markets suggests interdependence, whereas a contagion is present only in the case of a significant increase in co-movement.

Complementing what was mentioned earlier, we can add that markets are strongly interconnected, losses spread through them, and financial contagion is magnified [30]. This is because the fears of adverse market shocks tend to spread among investors, generating panic behavior [31]. Financial stress grows significantly driven by stock market volatility, high liquidity premiums, and contagion risks [32]. This finding is supported by [31], where emerging market shocks have statistically and economically significant impacts on global equity markets. Such a fact confirms the assumption of the initial systemic importance of emerging market economies as drivers of global asset price developments. Moreover, for a negative shock in a market, the probability of negative shocks in other markets increases [30].

The objective of this research is to statistically evaluate the financial effect generated by the health crisis derived from the COVID-19 pandemic on the performance of the economies belonging or not to the Organization for Economic Co-operation and Development (OECD). A statistical analysis of structural breakdown is conducted, considering the main stock market indexes by country, health index, and country risk. In addition, this research considers, from the financial point of view, a review of the literature associated with economic crises, development methodology, results, discussion, and conclusions.

The rest of the paper is organized as follows. In Section 2, the statistical model is formulated together with the methodology to be used. Furthermore, in this section, the stock indexes utilized are defined. Section 3 reports the results obtained from our investigation mentioning some indications and recommendations. In Section 4, we provide the conclusions, discussions, limitations, and further research of this study.

## 2. Modeling and Methodology

Models of structural breakdown associated with stock market indexes are widely used in the literature [31,33]. In addition, models that incorporate external effects, such as shock events, being them the main cause of the volatilities of the markets, have been also studied [30,32]. In order to carry out the statistical modeling, first, the structural breakdown in time series associated with the stock market indexes are calculated. This breakdown is assumed as a consequence of the measures taken by the different countries to control the mentioned shock events. These measures can affect the stock market value of the companies belonging to the different indexes. Then, the change in the level suffered by each stock index is measured and, with these measurements, regression models are formulated allowing us to define the impact of various factors on the structural change of economies [24,34,35].

**Figure 1:**
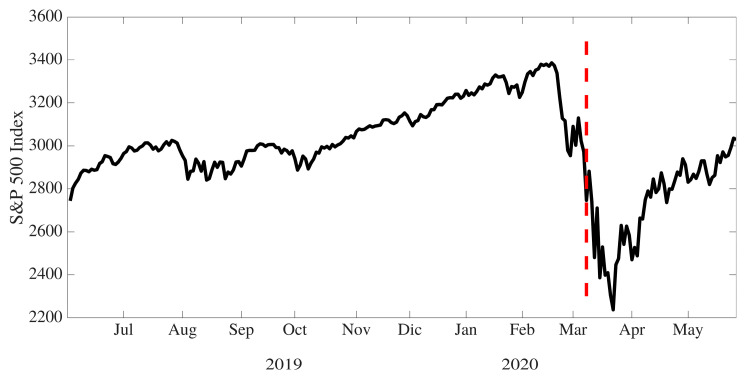
S&P500 price evolution from 1 June 2019 to 30 May 2020. The vertical line in red corresponds to the estimated date of structural breakdown (9 March 2020).

In this research, world stock market indexes are used to evaluate the evolution and performance of the economies of different countries during the periods both before and after the declaration of the global health crisis produced by the spread of COVID-19 [33,36], which can be considered as a shock event. As an example of structural breakdown associated with stock market indexes, Figure 1 shows the evolution of the S&P500 index, in which a structural breakdown in its price can be observed during the second week of March 2020.

The data used in the present study were obtained from Bloomberg at: https://bba.bloomberg.net/?utm_source=bloomberg-menu&utm_medium=bcom and from the GHS index at: https://www.ghsindex.org/wp-content/uploads/2019/10/2019-Global-Health-Security-Index.pdf. Data collection was made directly by each country for all of the series from the above-mentioned links. The indexes incorporated in this research are those that have the highest capitalization and volume in their markets. These indexes are the most traded and therefore the most liquid, allowing them to be representative of the entire analyzed universe [18]. The stock indexes considered here are based on daily observations of each time series; see Table 1. In this table, we describe the countries to be considered in the present study and their respective stock indexes. These countries (and their acronyms in parenthesis, when it corresponds) are Australia, Belgium, Brazil, Bulgaria, Canada, Chile, China, Colombia, Croatia, Cyprus, Egypt, Finland, France, Germany, Hungary, Iceland, India, Indonesia (IN), Iraq, Israel, Italy, Japan, Mexico, Malaysia, Nigeria, Norway, New Zealand (NZ), Pakistan, Peru, Philippines, Poland, Portugal, Qatar, Romania, Russia, Saudi Arabia (SA), Serbia, Slovakia, South Korea (SK), Spain, Sri Lanka (SL), Sweden, Switzerland (SW), Thailand, Turkey, Ukraine, United Kingdom (UK), and US. Within these countries, we consider cases with quite heavily COVID-19 hits, which is useful for our investigation.

By using the data collected for the stock indexes defined in Table 1, and based on the models supported by the background [30,31,32,33,36], we state a functional relation defined as

(1) $$Y_{i}=f\left(x_{1i},x_{2i},\;x_{3i},\;x_{4i},\;x_{5i}\right),$$

where $Y_{i}$ is the effect of the structural breakdown in country *i*; $x_{1i}=f_{1}\left({\mathrm{Health}}_{i}\right)$ is the value of *X*_1_ related to the health security of country *i* measured by the global health security (GHS) index for 2019 [37]; $x_{2i}=f_{2}\left({\mathrm{Risk}}_{i}\right)$ is the value of *X*_2;_ and $x_{3i}=f_{3}\left({\mathrm{StdRisk}}_{i}\right)$ is the value of *X*_3_, both of them associated with the average risk value and its standard deviation for country *i*, respectively; and two control variables $x_{4i}=f_{4}\left({\mathrm{OECD}}_{i}\right)$ is the value of *X*_4_, an indicator of whether or not country *i* belongs to the OECD group; as well as $x_{5i}=f_{5}\left({\mathrm{GDP}}_{i}\right)$ is the value of *X*_5_, which is linked to the gross domestic product (GDP) of country *i* [35]. Note that the response variable *Y* is measured as a percentage of the variation in the index between the average of the last three months (Diff3M), or six months (Diff6M), before the structural change and the value of the index as an average after two month of this change—which represents a loss of wealth of the countries present in this study; whereas Health represents a benchmarking of the health security of 195 countries [37].

We consider the period between July 2019 and May 2020 of the CDS on government bonds from execution to five years, provided by the Bloomberg database before mentioned.

Although CDS premiums do not capture exact default risk, the literature has documented that they are considered reliable and among the best default risk measures available [38]. Additionally, the CDS is used in [39] for risk analysis and as bond spreads because they are positively correlated with risk premium measures. However, the CDS show a higher correlation with country-specific credit risk drivers.

Based on Equation (1), the moment in which the structural breakdown of the series of indexes occurs for the different countries must be determined. In order to do this determination, the Wald test is used. This test consists of evaluating changes in the coefficients of a regression model during the periods defined by an unknown breakdown date, combined with the test statistics calculated for each possible breakdown date in the sample [40,41]. As mentioned, the period included for the analysis of each time series, described in Table 1, corresponds to 1 July 2019 until 28 May 2020. In this period, we work with 234 observations for 48 countries and indexes detailed in Table 2, which provides the estimated dates of structural breakdown using the Wald test.

From Table 2, note that the entire estimated structural breakdown corresponds to dates prior to 17 March 2020. However, there are three countries that appear atypical to this phenomenon, which are Iraq, SL, and Ukraine. Therefore, their structural breakdown can be caused due to a phenomenon other than the one studied in this paper [18].

**Figure 2:**
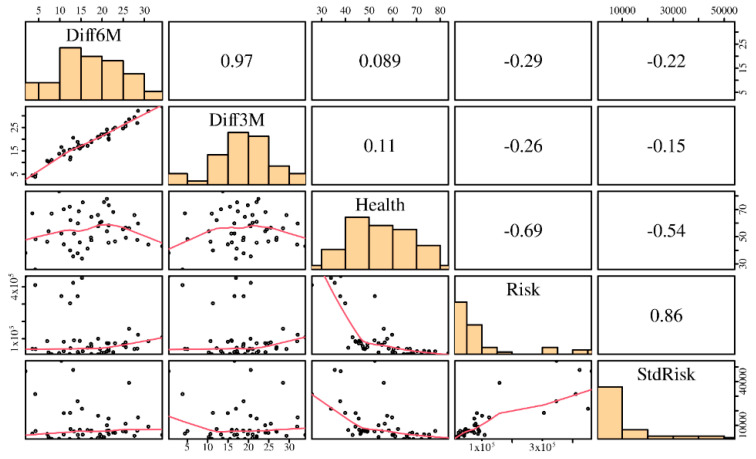
Descriptive summary of the variables studied.

According to [30], a negative shock in one market increases the probability of negative shocks in other markets. Methodologically, it is considered that when a disturbance occurs in one index, it is reflected in the modified dynamics of the other indexes [42,43]. The existence of a structural breakdown implies that the behaviour of the time series changes. Moreover, for all countries within this investigation, the null hypothesis of the non-existence of a structural breakdown is rejected, and the difference in the average of the index value is as previously described. The main descriptive statistics of the variables considered in the functional formulation defined in (1) are reported in Table 3, whereas the linear relationships between the studied variables are reported in Figure 2. In this figure, the upper triangular sector corresponds to the Pearson correlation of the indicated variables; the lower triangular part corresponds to the scatter plots between these variables; and the diagonal corresponds to the histogram of such variables.

From Figure 2, we detect adequate levels of correlation among the responses Diff3M and Diff6M with the covariates Health, Risk, and StdRisk, justifying the use of multiple linear regression models. However, when observing the correlations between the covariates Health, Risk, StdRisk, we assess a possible multicollinearity problem, which must be analyzed by means of the variance inflation factor (VIF). Its values for each estimated coefficient when modeling Diff3M are: VIF(Health) = 1.93, VIF(Risk) = 5.27, and VIF(StdRisk) = 3.91, whereas when modeling Diff6M, similar values are obtained. Note that all of the VIF values are less than 10, indicating no collinearity problems; see details about the VIF and the used criterion in [44].

In order to perform the estimation of the regression model parameters based on the functional formulation defined in (1), we establish statistical models for the response variable *Y* (Diff3M or Diff6M) in terms of the explanatory variables ($X_{1i},X_{2i},\;X_{3i},\;X_{4i},\;X_{5i}$), whose observed values are ($x_{1i},x_{2i},\;x_{3i},\;x_{4i},\;x_{5i}$), as well as of the variance $\left(\sigma_{i}^{2}\right)$ of the model error $\left(\epsilon_{i}\right)$, by means of

(2) $$Y_{i}=x_{i}\beta +\epsilon_{i},\;$$

(3) $$\sigma_{i}^{2}=\exp\left(z_{i}\alpha \right),$$

where $x_{i}$ corresponds to the vector of observed values of the explanatory variables denoted as $x_{1i}={\mathrm{Health}}_{i}$, $x_{2i}=\log\left({\mathrm{Risk}}_{i}\right)$, $x_{3i}=\log\left({\mathrm{StdRisk}}_{i}\right)$, $x_{4i}=$ 0 or 1 (depending if the country belongs to the OCDE or not), and $x_{5i}=\log\left({\mathrm{GDP}}_{i}\right)$ for country i. Note that $\beta =\left(\beta_{0},\;\beta_{1},\beta_{2},\beta_{3},\beta_{4},\beta_{5}\right)$ is a vector of regression parameters to be estimated [43,44].

In the formulation given in Equations (2) and (3), $\epsilon_{i}$ corresponds to the model error for observation i, which is assumed to be Gaussian distributed, centered on zero, and independent of the other error terms. In contrast, the variance of the error, represented by $\sigma_{i}^{2}$ in (3), is assumed to depend on control variables z, where $z_{i}$ corresponds to the vector of explanatory variables for country i and α is a vector of regression parameters to be estimated associated with these variables $z_{i}$.

Maximum likelihood (ML) and generalized least squares (GLS) estimators are compared with the ordinary least squares (OLS) estimator in terms of robustness [45]. If the form of the heteroscedasticity is correctly specified, then the ML and GLS estimators are more efficient statistically than the OLS estimator. Another alternative is using the generalized method of moments (GMM) to estimate the parameters. However, for relatively small samples, the GMM estimators are biased, as mentioned in [46,47,48].

For this research, the ML estimator is used to perform the data analysis. This is because the estimation by the ML method is more efficient when considering an adequate specification of the model, under the assumption that the error term is Gaussian distributed [49], and the sample is relatively small [50].

## 3. Results of the Study and Model Specification

The first two specifications, named Models 1 and 2, for the response variable *Y* (Diff3M or Diff6M) consider as explanatory variables: (i) health index; (ii) the logarithm of the average country risk measured through the CDS; and (iii) the logarithm of the standard deviation of the same country risk. These two specifications differ in that the average of the latter was considered when measuring structural change for three and six months of the evolution of the stock market indicators, respectively. The third and fourth specifications, named Models 3 and 4, consider as explanatory variables: (i) health index; (ii) the logarithm of the average country risk; (iii) the logarithm of its standard deviation; and (iv) an indicator of whether or not the country belongs to the OECD group. The fifth and sixth specifications, named Models 5 and 6, include as explanatory variables: (i) health index; (ii) the logarithm of average country risk; (iii) the logarithm of its standard deviation; (iv) an indicator of whether or not the country belongs to the OECD group; and (v) the logarithm of the GDP.

Table 4 reports the results of regression analyses for Models 1–6 of the size of the structural jump. For all cases, the regressions present non-significant parameters. We proceed to analyze the heteroscedasticity effect of the interaction that the different variables in the model can generate. In order to do this, the Breusch–Pagan test is applied to evaluate the heteroscedasticity effect in the regression model. For all models, the null hypothesis of no heteroscedasticity is rejected. Therefore, the phenomenon is modeled considering the effect of the different variables within the variance of the linear model.

Table 5 reports the linear regressions considering the effect of heteroscedasticity. As mentioned, Models 1 and 2 consider health index, logarithm of the average country risk and logarithm of the standard deviation of the same indicator of country risk. Similar to Table 2, they differ in that the structural change measurement was considered as the average of the last three and six months of the evolution of the stock market indicators, respectively. They also incorporate the same variables when modeling the variance. As also mentioned, Models 3 and 4 consider the same explanatory variables of Models 1 and 2, in addition to an indicator of membership to the OECD group. Models 5 and 6 are similar to Models 4 and 5, with the difference that, in the variance modeling, the logarithm of GDP is included.

Models 5 and 6 present significant parameters of the constant, whether or not they belong to the OECD group, the logarithm of Risk, logarithm of StdRisk, and Health. Models 2, 3 and 4 only present a high level of significance in the logarithm of StdRisk.

When analyzing the variance, both Models 5 and 6 indicate significant parameters associated with belonging or not to the OECD group, the logarithm of GDP, and the constant. Furthermore, Model 5 shows a high level of significance in the logarithms of Risk and StdRisk. In addition, note that Models 5 and 6 reject the null hypothesis that the coefficients defining the variance are equal to zero, which suggests that the model should not remove the variables associated with the variance for their adequate specification.

The linear part of the model shows that belonging to the OECD group has a positive impact on the size of the structural breakdown for Models 5 and 6. Therefore, belonging to the OECD group increases the effect of a structural change because these countries have greater trade openness and then a higher contagion effect on stock markets. In contrast, the significant and negative effects of both the Health and logarithm of the country risk indicate that a country with better institutional and economic conditions is less affected by a massive effect associated with the phenomenon of the pandemic if it had concrete effects on different stock markets.

From Table 4, note that, for Model 1, the mean percentage of loss of wealth of the studied countries increases in 0.0618 when the health index increases in one point. For Models 2–6, this increase is of 0.0199, 0.0312, 0.0125, 0.0243, and 0.0064, respectively. An analogous interpretation is obtained for the values presented in Table 5 corresponding to the explanatory variable health. Table 4 and Table 5 differ in considering homoskedasticity or heteroskedastic in the modeling, respectively.

It is worth mentioning that the effect of the health index of Models 5 and 6 has estimated values of its significant parameters. Thus, the health index used for this econometrical/statistical modeling determines the capacity of the different countries in responding efficiently when focusing a pandemic effect as viewed from the perspective of stock market developments. However, the contagion effect of stock markets cannot be ruled out as a preponderant factor when analyzing the structural breakdown that has occurred in the world economy.

## 4. Discussions, Conclusions, Limitations, and Future Research

After the recent global financial crises, and during the COVID-19 pandemic in particular—a crisis that is in full swing—appropriate models for describing events that lead to these crises have become quite essential in the areas of finance and risk management. Considering contagion as an equilibrium phenomenon that is generated from the fact that liquidity preference shocks are imperfectly correlated among regions, a financial crisis such as COVID-19 allows the term to capture the attention evaluating the negative financial repercussions for all nations.

Additionally, under normal circumstances, the stock market with its main indexes represents the economy of a country. However, the particular situation of COVID-19 must be considered. In this situation, the stock market does not necessarily represent the economy, because it depends on the contagion effect, the economic conditions, the interrelation among countries, and the development achieved by the companies that make up the main index.

Among the relevant results obtained from the structural breakdown analysis developed in this study, we reported that the estimated date of the structural breakdown for all of the series studied is between the last week of February 2020 and the third week of March 2020. This finding leads to the assumption that a common event was the generator of the global structural change, and we attribute it to the effect of COVID-19. In contrast, countries with better political and economic stability are less affected by the pandemic.

The effect of the health index, as an indicator of the response that countries may have when focusing a health crisis, shows that countries with better health conditions have less impact on the fall in stock indexes during health crisis as that caused by COVID-19.

We used a regression model with normal and heteroskedastic errors, which assumed that observations taken at different time points are mutually independent [11,12]. This assumption is a limitation of our proposal and could be improved by the search for breakdown points in time series, as done in [51] and literature therein.

The determination of the breaking point of the world financial indexes, based on the analysis of the main indexes considered in the present study, allows us to conclude that this breaking point is attributed to the financial contagion effect of the markets that generates such a break. Therefore, independent of the health index that is determined for a country, the contagion effect is preponderant at crisis times. This allows a prediction of the independent market that affects a country from a health point of view.

This investigation can be improved by considering longer times especially when the virus has ended. This can be possible by analyzing specific geographical areas [52], and by assuming variables and indexes that have recovered faster in the global financial markets to understand the phenomenon from a better perspective.

An important aspect to be further studied is to measure the efficiency and impact of the relevant variables and factors on the stock markets [53,54]. Extensions to the multivariate case [55] and the incorporation of temporal [56], spatial [52,57], and quantile regression [57] structures in the modeling, as well as errors-in-variables [58], and PLS regression [44], are also of practical relevance.

The derivation of diagnostic techniques [55] to detect potential influential cases are needed, which are an important tool to be used in all statistical modeling, as well as the use of robust estimation methods [59] are also of empirical interest.

Therefore, the methodology proposed in this investigation promotes new challenges and offers an open door to explore other theoretical and numerical issues. Research on these and other issues are in progress and their findings will be reported in future articles.

## Figures and Tables

**Table 1:** Stock index by country used in the study (* EN: Euronext; ** MT: Msci Tadawul).

Country	Index	Country	Index	Country	Index	Country	Index
Australia	Asx 200	France	* EN 100	Nigeria	Nse 30	Serbia	Belex 15
Belgium	Bel 20	Germany	Dax	Norway	Oseax	Slovakia	Sax
Brazil	Ibov	Hungary	Bux	NZ	Nzx 50	SK	Kospi 50
Bulgaria	Sofix	Italy	Ftse Mib	Pakistan	Kse 100	Spain	Ibex
Canada	Tsx	India	Nifty 50	Peru	Igbvl	SL	Cse
Chile	Ipsa	IN	Jci	Philippines	Psei	Sweden	Omx 30
China	Shanghai	Irak	Isx	Poland	Wig 30	SW	Smi
Colombia	Colcap	Iceland	Icexi	Portugal	Psi 20	Thailand	Set
Croatia	Crobex	Israel	Ta 100	Qatar	Qe	Turkey	Xu 100
Cyprus	Cymain	Japan	Nikkei 225	Romania	Bet	Ukraine	Pfts
Egypt	Egx 70	Malaysia	Fbm Klci	Russia	Moex	UK	Ftse 100
Finland	Hex 25	Mexico	Mexbol	SA	** MT 30	US	S&P500

**Table 2:** Structural breakdown date estimated by the Wald test for the indicated country.

Country	Date	Country	Date	Country	Date	Country	Date
Australia	03/09/20	France	03/09/20	Nigeria	03/11/20	Serbia	03/12/20
Belgium	03/09/20	Germany	03/09/20	Norway	03/09/20	Slovakia	03/17/20
Brazil	03/04/20	Hungary	03/09/20	NZ	03/12/20	SK	03/12/20
Bulgaria	03/09/20	Italy	03/09/20	Pakistan	03/16/20	Spain	03/09/20
Canada	03/09/20	India	03/12/20	Peru	03/12/20	SL	01/31/20
Chile	03/13/20	IN	03/09/20	Philippines	03/09/20	Sweden	03/09/20
China	03/16/20	Iraq	12/18/19	Poland	03/09/20	SW	03/06/20
Colombia	03/09/20	Iceland	03/06/20	Portugal	03/09/20	Thailand	03/09/20
Cyprus	03/09/20	Israel	03/09/20	Qatar	03/02/20	Turkey	03/09/20
Croatia	03/09/20	Japan	03/06/20	Romania	03/09/20	Ukraine	02/10/20
Egypt	03/16/20	Malaysia	03/06/20	Russia	03/10/20	UK	03/09/20
Finland	03/09/20	Mexico	03/09/20	SA	03/09/20	US	03/09/20

**Table 3:** Descriptive statistics of the variables considered in the model.

Variable	Minimum	Maximum	Mean	Standard Deviation
Health	25.80	83.50	54.87	13.22
Risk	11,037.89	462,453.50	106,106.13	130,742.35
StdRisk	505.46	54,134.31	10,521.88	12,775.89
Diff3M	1.87	34.23	16.67	7.69
Diff6M	0.47	33.96	18.34	7.44

**Table 4:** Parameter estimate and the corresponding standard error (in parenthesis) of the indicated model, as well as statistical indicators of goodness-of-fit ($R^{2})$ and significance.

	Model 1	Model 2	Model 3	Model 4	Model 5	Model 6
Variable	(Diff3M)	(Diff6M)	(Diff3M)	(Diff6M)	(Diff3M)	(Diff6M)
Health	0.0618	0.0199	0.0312	0.0125	0.0243	0.0064
	(0.141)	(0.145)	(0.149)	(0.154)	(0.150)	(0.155)
Log(Risk)	−1.631	−2.574	−1.477	−2.537	−0.61	−1.773
	(3.026)	(3.111)	(3.056)	(3.156)	(3.239)	(3.353)
Log(StdRisk)	1.712	2.146	1.719	2.148	0.923	1.447
	(2.215)	(2.276)	(2.23)	(2.303)	(2.433)	(2.519)
OECD			1.806	0.437	1.36	0.0449
			(2.766)	(2.857)	(2.827)	(2.927)
Log(GDP)					0.463	0.408
					(0.556)	(0.576)
Constant	18.37	25.6	17.36	25.35	12.49	21.06
	(27.09)	(27.85)	(27.32)	(28.22)	(28.04)	(29.03)
$R^{2}$	0.026	0.028	0.035	0.029	0.051	0.040
F-statistic	0.376	0.415	0.385	0.31	0.444	0.345
**Breusch** **–** **Pagan test**						
$\chi^{2}$(1)	4.10	3.92	7.10	4.60	5.29	3.68
Prob $>\chi^{2}$	0.0428	0.0476	0.0077	0.0320	0.0214	0.0551

* *p* < 0.1, ** *p* < 0.005, *** *p* < 0.001.

**Table 5:** Parameter estimate and standard error of the indicated model considering heteroscedasticity, as well as statistical indicators of goodness-of-fit ($R^{2})$ and significance.

	Model 1	Model 2	Model 3	Model 4	Model 5	Model 6
Variable	(Diff3M)	(Diff6M)	(Diff3M)	(Diff6M)	(Diff3M)	(Diff6M)
Health	0.038	0.0203	−0.00258	0.00543	−0.130 ***	−0.130 ***
	(0.113)	(0.118)	(0.104)	(0.116)	(0.0190)	(0.0275)
Log(Risk)	−1.545	−2.903	−1.811	−3.105	−3.628 ***	−5.336 ***
	(2.744)	(2.946)	(2.588)	(2.900)	(0.403)	(0.584)
Log(StdRisk)	2.481	3.535 *	3.114 *	3.900 **	4.718 ***	6.035 ***
	(1.877)	(2.010)	(1.718)	(1.897)	(0.266)	(0.386)
OECD			3.367	1.924	8.157 **	7.115 *
			(2.325)	(2.47)	(3.44)	(3.796)
Constant	12.33	17.32	9.765	16.69	21.20 ***	26.65 ***
	(22.9)	(24.7)	(21.14)	(24.30)	(4.992)	(6.518)
$\mathrm{Log}\left(\sigma^{2}\right)$						
Health	−0.018	−0.0244	−0.0133	−0.0165	0.00280	0.000176
	(0.0261)	(0.0256)	(0.0261)	(0.0261)	(0.0401)	(0.0441)
Log(Risk)	0.761	0.723	0.915	0.833	−1.905 *	−1.545
	(0.586)	(0.575)	(0.624)	(0.608)	(1.055)	(1.083)
Log(StdRisk)	−0.342	−0.439	−0.608	−0.566	1.549 **	1.291
	(0.472)	(0.445)	(0.493)	(0.462)	(0.780)	(0.786)
OECD			−0.847 *	−0.627	−1.532 **	−1.235 *
			(0.467)	(0.454)	(0.714)	(0.716)
Log(GDP)					−2.253 ***	−2.136 ***
					(0.568)	(0.550)
Constant	−0.598	1.117	0.103	0.851	25.78 ***	23.13 **
	(4.978)	(4.997)	(4.918)	(4.958)	(8.948)	(9.030)
**Likelihood Ratio Test for** $\mathrm{Log}\left(\sigma^{2}\right)=0$						
$\mathrm{Prob}>\chi^{2}$	0.0570	0.1180	0.0144	0.0824	0.0000	0.0001

* *p* < 0.1, ** *p* < 0.005, *** *p* < 0.001.

## Data Availability

The data used to support the findings of this study are available from the corresponding author upon request.
